# Increased Neck Circumference and Increased Waist-Hip Ratio: Predictive Factors of Acute Myocardial Infarction

**DOI:** 10.7759/cureus.36777

**Published:** 2023-03-28

**Authors:** Muhammad S Jibran, Muhammad Suleman, Shafi Ullah Khan

**Affiliations:** 1 Department of Cardiology, Medical Teaching Institute, Lady Reading Hospital, Peshawar, PAK; 2 Department of Cardiology, Mufti Mehmood Memorial Teaching Hospital, Dera Ismail Khan, PAK; 3 Department of Cardiology, Peshawar Institute of Cardiology, Peshawar, PAK; 4 Department of Medicine, District Headquarter Hospital KDA (Kohat Development Authority), Kohat, PAK

**Keywords:** acs ( acute coronary syndrome ), cardiovascular diseases, waist-hip ratio, obesity, neck circumference

## Abstract

Background

We hypothesize that neck circumference (NC) is a better predictor of acute myocardial infarction (AMI) compared to the waist-hip ratio (WHR) in patients presenting with acute coronary syndrome (ACS). The objective of this study is to investigate the association between NC and WHR with AMI and determine whether NC is a superior predictor of AMI in ACS patients compared to WHR.

Methods

This cross-sectional observational study was conducted in the Department of Cardiology at the Medical Teaching Institute, Lady Reading Hospital, Peshawar. The study lasted from February 20, 2018, to September 12, 2018. Patients having ACS who presented to the emergency department were enrolled via non-probability convenient sampling. Demographic data and baseline variables, including NC and WHR, were documented using a pre-designed pro forma. SPSS V.20 (IBM Corp, Armonk, NY) was used for data analysis. Continuous variables were expressed as mean ± standard deviation, while categorical variables were presented as frequencies and percentages. Chi-square tests were performed to determine the association between variables, and logistic regression models were used to measure odds ratios (ORs).

Results

In this study, 180 patients were included, with a mean age of 54.48±8.48 years and a male predominance of 51.5%. The results indicated a significant association between increased NC and WHR with AMI. The chi-square values for NC and WHR were 78.26 (p≤0.001) and 43.38 (p≤0.001), respectively. As NC increased from <37 cm to >38.5 cm, the OR for AMI increased from 0.46 to 4.51. Furthermore, the prevalence odds ratio (POR) of AMI increased by 2.185 times with an increase in WHR from 0.90.

Conclusion

Increased NC and increased WHR are statistically significantly associated and strong predictors of AMI in ACS patients. However, NC being more reliable, effective, and user-friendly should be the preferred measure.

## Introduction

Coronary artery disease (CAD) contributes significantly to morbidity and mortality worldwide [1]. The increasing industrialization of developing countries has led to rapid demographic, economic, geographical, and environmental changes, resulting in a rise in the disease burden of CAD globally. CAD is a common cause of hospital admissions in Pakistan, accounting for approximately 16.4% [2]. 

Obesity, particularly fat deposition in the upper body, has been identified as a well-established risk factor for developing metabolic syndrome and CAD, diabetes mellitus, and hypertension [3,4]. Recent research has emphasized the paracrine effect of adipose tissue, which can significantly impact triglyceridemia, insulinemia, and uric acid levels in the body, thereby aggravating the pathophysiology of CAD [5].

Several studies have indicated that neck circumference (NC), a measure of upper body fat, is strongly associated with metabolic syndrome, dysglycemia, and non-alcoholic fatty liver disease (NAFLD), irrespective of the patient's body mass index [6]. Upper-body fats, mainly subcutaneous fats, have been reported to correlate more strongly with insulin resistance, obesity, diabetes mellitus, and cardiovascular disease (CVD) than lower-body fats. It has gained the particular interest of researchers recently [7]. One centimeter increase in NC increases the 10-year risk of CVD by 3 % in men and 5 % in women, and the risk increases many folds for NC above 40 cm, up to 18% in men and 35% in women [8]. NC has been reported as an independent significant risk factor and marker for subclinical atherosclerotic cardiovascular disease (ASCVD) [9]. Increased NC has also been correlated with increased markers of inflammation (interleukin 6 [IL-6], tumor necrosis factor-alpha [TNF-alpha], C3, C4) and endothelial dysfunctions (E-selectin) [10]. Increased NC has also been associated with an increased risk of obstructive sleep apnea, the leading risk factor for ASCVD and diabetes mellitus [11].

The primary aim of this study is to explore the association between acute myocardial infarction (AMI) and anthropometric measurements, specifically NC and waist-hip ratio (WHR), in patients seeking medical care at a tertiary healthcare center for acute coronary syndrome (ACS). In addition, this research aims to evaluate whether NC is a superior predictor of AMI compared to WHR. Although previous studies have established a connection between NC and various risk factors, such as obesity, metabolic syndrome, insulin resistance, and insulin clearance, there is still a gap in understanding its potential to predict cardiovascular events as a composite outcome [12]. This study seeks to fill this knowledge gap.

## Materials and methods

This observational study was performed cross-sectionally in the Department of Cardiology, Medical Teaching Institute, Lady Reading Hospital, Peshawar, between February 20, 2018, and September 12, 2018. Ethical approval was taken from the Institutional Review Board of the same hospital. Patients who presented in the emergency department with ACS meeting the inclusion and exclusion criteria were enrolled using non-probability convenient sampling. ACS was defined by chest pain or discomfort, shortness of breath, sweating, nausea, vomiting, palpitations, or other signs of myocardial ischemia, accompanied by electrocardiography changes and/or elevation of cardiac biomarkers. AMI was defined as a patient having either ST-elevation myocardial infarction (STEMI) or non-ST-elevation myocardial infarction (NSTEMI). Unstable angina (UA) was defined as all patients with ACS who had negative serial troponin T results. 

Inclusion and exclusion criteria

Patients from both genders with ages >25 years and having a presentation of ACS were included in the study. Patients with a history of STEMI, prior coronary interventions, cardiomyopathy of all types, cardiac failure with New York Heart Association (NYHA) III and above, estimated glomerular filtration rate less than 30, neck deformities, thyroid problems, Cushing syndrome, and NC of extreme measurements (less than 25 cm and above 60 cm) were excluded from the study. 

Data collection 

The baseline characteristics of the patients were recorded, and NC was measured at the cricoid cartilage using an elastic tape. The patients were divided into three categories based on their NC measurements: NC <37 cm, 37-38.5 cm, and >38.5 cm. The waist and hip circumferences were also measured using elastic tape, and the ratios were calculated. According to the WHO criteria, a WHR >0.9 was considered a marker of obesity. All data were recorded in a pre-designed pro forma. The patients were subdivided into AMI and UA based on troponin T levels. 

Statistical analysis

The statistical software SPSS version 20 (IBM Corp, Armonk, NY) was used to analyze the collected data. Continuous variables were presented as mean ± standard deviation, while categorical variables were presented as frequencies and percentages. The association between NC and AMI as well as elevated WHR and AMI was assessed using the chi-square test. To evaluate the predictive ability of NC and increased WHR for AMI, logistic regression models were constructed. Statistical significance was set at p <0.05.

## Results

 A total of 180 study participants, with a mean age of 54.48±8.48 years, were enrolled, among whom 51.5% were male and the remaining were female. Table 1 presents the baseline characteristics, including demographic variables and comorbidities.

**Table 1:** Baseline Characteristics of Study Subjects (n=180) CAD, coronary artery disease; WHR, waist-hip ratio.

Variables		Mean ± SD	Frequency	Percentage
Age (years)		58.48±8.48	---	---
Male		---	92	51.5
Female		---	88	48.5
Hypertension		---	109	60.6
Smoking		---	76	42.2
Diabetes mellitus		---	74	41.1
Insulin		---	38	21.1
Oral hypoglycemics		---	36	20
Family history of CAD		---	60	33.3
Neck circumference (cm)		38.57±2.41	---	---
Neck circumference categories	<37 cm	---	41	22.8
	37-38.5 cm	---	55	30.6
	>38.5 cm	---	84	46.7
Hip circumference (cm)		98.61±13.25	---	---
Waist circumference (cm)		91.44±14.41	---	---
WHR		0.90±0.09	---	---
Raised WHR (>0.9)		---	108	60
Acute coronary syndrome		---	180	100
Unstable angina		---	73	40.6
Acute myocardial infarction		---	107	59.4
ST-elevation myocardial infarction		---	70	38.9
Non-ST-elevation myocardial infarction		---	37	20.6
Cholesterol (mg/dl)		199.18±25.07	---	---
Triglycerides (mg/dl)		185.58±4.82	---	---
High-density lipoprotein (mg/dl)		40.39±4.5	---	---
Low-density lipoprotein (mg/dl)		123.51±28.8	---	---

The association between raised NC and AMI and raised WHR and AMI was determined in cross-tab using the chi-square test as shown in Table 2.

**Table 2:** Association of NC and WHR with AMI NC, neck circumference; WHR, waist-hip ratio; AMI, acute myocardial infarction; ACS, acute coronary syndrome.

	Neck circumference (in cm)			
AMI	<37	37-38.5	>38.5	Total
Yes	3	29	75	107
No	38	26	9	73
Total	41	55	84	180
Chi-square value for neck circumference vs ACS: 78.26 (p=0.001)				
	Waist-hip ratio			
AMI	Normal	>0.9	Total	
Yes	21	86	107	
No	50	23	73	
Total	71	109	180	
Chi-square value for waist-hip ratio vs ACS: 43.38 (p=0.001)				

Here it is evident that X2-value for raised NC is 78.26 compared to raised WHR, which is 43.38. Though both values are statistically significant, raised NC is more closely associated with AMI.

The odds ratio (OR) of developing AMI in ACS patients was determined in various NC categories, and it was found that with increasing NC, the prevalence odds ratio (POR) for AMI increased. Also, the POR was determined for raised WHR by using a logistic regression model, as shown in Table 3.

**Table 3:** POR for increasing NC categories and increasing WHR for AMI POR, prevalence odds ratio; NC, neck circumference; WHR, waist-hip ratio; AMI, acute myocardial infarction.

Variable	Acute myocardial infarction				
NC category	POR	Exp(B)	p-Value	Confidence interval (Exp-B)	
<37 cm	0.463	0.436	0.001	0.219	0.739
37-38.5 cm	2.241	1.312	0.001	0.681	1.987
>38.5 cm	4.512	1.703	0.001	0.834	2.103
Normal WHR	0.781	0.739	0.001	0.431	1.011
Raised WHR	2.185	1.310	0.001	0.981	1.737

## Discussion

Among the various contributing factors to CVD, the regional distribution of body fat has emerged as a significant risk factor. Traditional measures of central obesity, such as waist and hip circumferences, have been employed to assess the association between fat distribution and CVD. However, NC measurement is a new way to measure cervical obesity and is reported to be as good as WHR in a study with cutoff values of 32 cm for women and 37 cm for men to predict the risk of ASCVD [13]. 

In contrast, lower-body fat, particularly in the hip and thigh regions, may protect against ectopic fat accumulation. Ectopic fat has been linked to the development of insulin resistance, reduced insulin clearance, metabolic syndrome, and NAFLD, which can progress to cirrhosis. Furthermore, studies have reported its association with cancer and CAD [14]. 

The literature has reported the correlation between epicardial fat and mortality in CAD [15]. More recent studies have suggested that perivascular, perirenal, pericardial, liver, and muscle fat can also contribute to the development of CAD [16,17]. It has been observed that the release of free fatty acids by upper-body fats is higher than that of lower-body fats [18]. Although NC has gained attention as a research area for its association with obesity and CAD risk, its predictive ability for ACS has not yet been evaluated. One study indicated that increased NC was associated with higher heart failure hospitalization rates and had prognostic value in predicting composite outcomes of ischemic heart disease, particularly heart failure hospitalization [19]. 

Comorbidity of diabetes mellitus further increases the risk of cardiovascular outcomes by two to three times (HR=2.305, all confounders adjusted) in patients with increased NC (>43 cm in men and >39 cm in women) [12].

Our study determined the association between NC and AMI and found an association between NC and raised WHR. We found that NC can predict the incidence of AMI in patients with ACS. Our analysis showed a significant association between raised NC and AMI, with a chi-square value of 78.26 (p-value: 0.001). In contrast, the association between increased WHR and AMI had a chi-square value of 43.38 (p-value: 0.001). Although both values were statistically significant, increased NC had a closer association in AMI patients. Additionally, we found that the probability of having AMI increased in ACS patients from 0.463 to 2.24 and 4.51 times when the NC increased from <37 cm to 37-38.5 cm to >38.5 cm, respectively. We also found an association with other anthropometric measures like WHR and waist and hip circumferences.

The NC measurement presents a convenient, easily measurable, and reliable alternative with less margin of error than the WHR measurement, which may be influenced by various factors such as meals, clothing, and respiration [20]. NC measurement is a low-cost, simple, and easily accessible tool that holds the potential to predict AMI in patients presenting with ACS. Our study examined the correlation between NC and AMI and assessed the impact of elevated WHR as an indicator of obesity. 

This study is unique in that it is the first to evaluate the odds of AMI associated with raised NC in a sub-continental population, highlighting its strength and novelty. It also brings the concept of cervical obesity, which is new to our population and needs to be adapted at the primary level. 

The primary constraint of our study was its dual-center design, cross-sectional type, and non-randomization, which restricted the generalizability of the findings beyond the geographic regions of the selected study sites located in Khyber Pakhtunkhwa province, Pakistan. Further, following these cohorts prospectively is suggested at the provincial and country levels to see their correlation with CAD, as reported by one study of an eight-year cohort in China [21].

## Conclusions

The findings of the study reveal that both NC and WHR have a significant statistical association with AMI in patients presenting with ACS, and can also predict the OR of developing this condition. However, when considering the practicality and accessibility of these measurements, NC emerges as a more user-friendly option compared to WHR. In particular, the simplicity and ease of measuring NC make it a preferable choice for predicting the risk of myocardial infarction in clinical settings. As such, health professionals can benefit from incorporating NC as a routine measurement in their assessments for identifying patients at a higher risk of developing AMI. Overall, this study highlights the importance of using convenient and accessible methods to accurately identify individuals who may be at a greater risk of developing CVDs such as AMI.
